# Antibiotic fidaxomicin is an RdRp inhibitor as a potential new therapeutic agent against Zika virus

**DOI:** 10.1186/s12916-020-01663-1

**Published:** 2020-07-31

**Authors:** Jie Yuan, Jianchen Yu, Yun Huang, Zhenjian He, Jia Luo, Yun Wu, Yingchun Zheng, Jueheng Wu, Xun Zhu, Haihe Wang, Mengfeng Li

**Affiliations:** 1 Department of Biochemistry, Zhongshan School of Medicine, Sun Yat-sen University, Guangzhou, 510080 China; 2 Key Laboratory of Tropical Disease Control (Sun Yat-sen University), Ministry of Education, Guangzhou, 510080 China; 3 Cancer Institute, Southern Medical University, Guangzhou, 510515 Guangdong China; 4 Department of Microbiology, Zhongshan School of Medicine, Sun Yat-sen University, Guangzhou, 510080 China; 5 School of Public Health, Sun Yat-sen University, Guangzhou, 510080 Guangdong China; 6 School of Pharmacy, Guangdong Pharmaceutical University, Guangzhou, 510006 China; 7 Southern Laboratory of Ocean Science and Engineering (Zhuhai), Zhuhai, 519000 Guangdong China; 8 School of Life Science and Biopharmaceutics, Guangdong Pharmaceutical University, Guangzhou, 510006 China

**Keywords:** Zika virus, Fidaxomicin, RdRp inhibitor, NS5, Antiviral

## Abstract

**Background:**

Zika virus (ZIKV) infection is a global health problem, and its complications, including congenital Zika syndrome and Guillain-Barré syndrome, constitute a continued threat to humans. Unfortunately, effective therapeutics against ZIKV infection are not available thus far.

**Methods:**

We screened the compounds collection consisting of 1789 FDA-approved drugs by a computational docking method to obtain anti-ZIKV candidate compounds targeting ZIKV RNA-dependent RNA polymerase (RdRp). SPR (BIAcore) assay was employed to demonstrate the candidate compounds’ direct binding to ZIKV RdRp, and polymerase activity assay was used to determine the inhibitory effect on ZIKV RdRp-catalyzed RNA synthesis. The antiviral effects on ZIKV in vitro and in vivo were detected in infected cultured cells and in *Ifnar1*^*−/−*^ mice infected by ZIKV virus using plaque assay, western blotting, tissue immunofluorescence, and immunohistochemistry.

**Results:**

Here, we report that a first-in-class macrocyclic antibiotic, which has been clinically used to treat *Clostridium difficile* infection, fidaxomicin, potently inhibits ZIKV replication in vitro and in vivo. Our data showed that fidaxomicin was effective against African and Asian lineage ZIKV in a wide variety of cell lines of various tissue origins, and prominently suppressed ZIKV infection and significantly improved survival of infected mice. In addition, fidaxomicin treatment reduced the virus load in the brains and testes, and alleviated ZIKV-associated pathological damages, such as paralysis, hunching, and neuronal necrosis in the cerebra. Furthermore, our mechanistic study showed that fidaxomicin directly bound ZIKV NS5 protein and inhibited the RNA synthesis-catalyzing activity of ZIKV RdRp.

**Conclusions:**

Our data suggest that fidaxomicin may represent an effective anti-ZIKV agent. In the light that fidaxomicin is already a clinically used drug, there might be a promising prospect in the development of fidaxomicin to be an antiviral therapeutic.

## Background

Zika virus (ZIKV) is a member of the *Flaviviridae* family first isolated in 1947 from a sentinel rhesus macaque in the Zika Forest region in Uganda [1]. For the following 60 years, only scattered cases were identified in Africa and Asia until 2007, when ZIKV emerged in a series of outbreaks across the Pacific [2–5]. A large pandemic expanded in 2014 and 2015 when ZIKV exploded into the Americas dramatically [6–8]. By now, ZIKV infections have widespread in the Americas and Southeast and South Asia and have become a threat to all tropical and temperate countries. The World Health Organization declared the clusters of microcephaly and neurological disorders and their association with ZIKV infection to be a global public health emergency, highlighting the significance and severity of this virus in human beings [9].

Most flaviviruses are transmitted by mosquitos; however, ZIKV can also spread directly through sexual contact [10–13] and vertically from mother to fetus [14–16]. In general, ZIKV infection is mostly asymptomatic, and most symptomatic infections are mild and resemble those observed with dengue viral infection presenting symptoms and signs of rash, fever, arthralgia, conjunctivitis, myalgia, headache, and retro-orbital pain [17]. However, infection of ZIKV has been strongly associated with not only neurological sequelae, most commonly Guillain-Barré syndrome (GBS) [18], but also meningo-encephalitis and myelitis [19]. Notably, when the infection occurs during pregnancy, severe fetal abnormalities and death maybe the most serious complications of the virus infection, which underscore a particular importance distinct from other pathogenic flaviviruses [20, 21].

Despite diversity in pathogenesis, the *Flavivirus* members have highly similar viral structures and genomic organization. Like other flaviviruses, ZIKV is an enveloped virus with a positive-sense, single-stranded RNA genome, which encodes a single open reading frame (ORF). Translation of the long ORF produces a large polyprotein which is then cleaved by both viral and host proteases to generate ten individual viral proteins, which include three structural proteins (capsid, prM, and E) and seven non-structural proteins (NS1, NS2A, NS2B, NS3, NS4A, NS4B, and NS5) [22].

It is noteworthy that effective therapeutics of ZIKV are not currently available [23]. Developing drugs targeting viral proteins key to the replication represents a potentially promising strategy for the treatment of ZIKV infection, as it is also pursued in other flaviviridae diseases such as hepatitis C virus (HCV) and dengue virus (DENV) infections [24, 25]. Therefore, efforts in identifying viral target(s) and compounds targeting these targets are urgently needed.

In the context of finding ZIKV protein targets for treatment, it is of great interest to note that the RNA-dependent RNA polymerase (RdRp) has been identified as a potentially targetable component for anti-flavivirus drugs [26]. Specifically, RdRp is known as an enzyme essential for the replication of flaviviral RNA genome, which is key to the multiplication of flaviviruses, whose failure leads to cessation of the viral life cycle [27]. Moreover, the catalytic center of RdRp is relatively conserved among flaviviruses but absent in eukaryotic host cells, presumably providing a relatively safety profile for the antivirals in this drug category [28]. In addition, RdRp is a domain of NS5, and the latter catalyzes multiple biochemical reactions and interacts with numerous regulators, such as interferon suppression by targeting the IFN-regulated transcriptional activator STAT2 for degradation [29, 30], providing potential of targeting ZIKV NS5 for antiviral purposes. Furthermore, RNA polymerase inhibitors, such as sofosbuvir [31], ribavirin [32], beclabuvir [33], and favipiravir [34], have been clinically tested with success as antivirals approved for the treatment of viral infections [35], although no drug has been approved yet for specific antiviral treatment of flaviviral diseases. Taken together, RdRp inhibition might represent a potentially promising anti-ZIKV strategy.

The RdRp domain is at the C-terminus of the ZIKV NS5, which also contains a methyltransferase (MTase) domain at the N-terminus. The NS5 protein is the largest ZIKV protein (approximately 100 kDa), and its structure is highly conserved among the members of the *Flaviviridae* family, including ZIKV, DENV, and Japanese encephalitis virus (JEV) [28, 36]. As previous studies indicate that protein structural conservation might be associated with a functional consistency, multiple alignment of amino acid residues of ZIKV NS5 and its flaviviral homologs may provide a foundation for the uncovering of binding pocket(s) for effective inhibitory binding by inhibitors of RdRp. Moreover, the crystal structures of the ZIKV full-length NS5 protein and RdRp domain have been reported [27, 36–39]. The ZIKV RdRp has a typical viral RdRp structure, which adopts a classic “right-hand” structure consisting of the palm, finger, and thumb subdomains. The rich structural details should inform in silico and functional screening of inhibitors against ZIKV RdRp and accelerate the identification of effective anti-ZIKV agents. In this paper, we identified a first-in-class macrocyclic antibiotic fidaxomicin, which is being used clinically to treat *Clostridium difficile* infection (CDI) [40], as an effective antiviral agent against ZIKV. Herein, we show that fidaxomicin directly bound ZIKV RdRp, inhibited ZIKV RNA replication in vitro and in vivo, and suppressed ZIKV-induced disease in infected mice.

## Methods

### Cell culture, virus, and animals

Human lung carcinoma cell line A549, human hepatocellular carcinoma cell line Huh7, African green monkey kidney epithelial cell line Vero, human embryonic kidney cell line 293T, and human umbilical vein endothelial cells (HUVECs) were obtain from the Cell Bank of the Chinese Academy of Sciences (CBCAS), Shanghai, China. Human glioblastoma cell line SNB19 (CRL-2219, ATCC, Manassas, VA, USA) and human glioblastoma cell line A172 (ATCC CCL-1620) were obtained from ATCC. A549, Huh7, Vero, 293T, SNB19, and A172 cells were cultured in DMEM (Invitrogen, Carlsbad, CA, USA) containing 10% fetal bovine serum (FBS) (GIBCO, Carlsbad, CA, USA), 2 mM l-glutamine, 100 μg/mL streptomycin, and 100 units/mL penicillin (Invitrogen) at 37 °C under 5% CO_2_. HUVECs were grown in M199 medium (GIBCO, Carlsbad, CA, USA) supplemented with 15% (vol/vol) FBS, 2 mM l-glutamine, 0.02 mg/mL endothelial cell growth supplement (Corning, NY, USA), and 0.05 mg/mL heparin (Sigma-Aldrich, St. Louis, MO, USA). *Aedes albopictus* C6/36 cells (ATCC, CRL-1660) were maintained at 28 °C with 5% CO_2_ in DMEM supplemented with 10% FBS. All cell lines were authenticated by short tandem repeat (STR) DNA profiling and verified to be mycoplasma-free. ZIKV ZG-01 strain (China, 2016, GenBank accession number KY379148) and MR766 strain (Uganda, 2016, GenBank accession number MK105975), respectively, were kind gifts from professor Xi Huang and professor Hui Zhang (Sun Yat-sen University, Guangzhou, China) [41, 42]. Both virus stocks were grown in C6/36 cells and quantified by plaque assay. The Dengue-2 virus New Guinea C strain (GenBank accession number M29095) was provided by Guangzhou Centers for Disease Control and propagated in C6/36 cells [43]. Type-I interferon receptor-deficient (*Ifnar1*^−/−^) mice with a C57BL/6 background were provided by professor Gucheng Zeng and bred in specific pathogen-free animal facilities at Sun Yat-sen University [44]. Mice approximately 5 weeks of age were used for these experiments. For ZIKV infection and antiviral treatment studies, mice were randomly assigned to groups of 10 animals each to ensure the adequate power.

### Compounds database

The compounds database used for in silico screening was supplied by courtesy of Target Molecule Corp. (TargetMol, http://targetmol.com/). The collection consisted of a dataset of 1789 drugs approved by the Food and Drug Administration (FDA) with the information of chemical structure and biological activities.

### Chemicals and nucleotides

Fidaxomicin (catalog no.: HY-17580, batch no.: 15276) and ribavirin (catalog no.: HY-B0434, batch no.: 11970) were supplied in powder form by MedChemExpress LLC (Shanghai, China) together with quality control documents. Fluorescently labeled RNA oligonucleotides (Cy5-RNA) as well as unlabeled RNA and DNA oligonucleotides were chemically synthesized and HPLC purified by RiboBio Co., Ltd. (Guangzhou, China). Cy5-RNA primer sequences are shown in Fig. 2f.

### Plasmids

cDNAs coding for ZIKV NS5 protein and its RdRp domain (aa271~903), both with N-terminal 8× histidine tags, were PCR amplified from ZG-01 strain viral cDNA as a template and cloned into pcDNA3.1 or pET-30a-mutant vectors. pISRE (IFN-stimulated response element)-luciferase reporter construct (pISRE-Luc) was based on the pGL3-Promoter Vector, according to a previously described method [45]. All constructs were verified by DNA sequencing. All primers used for plasmid construction are listed in Additional file 1: Table S1.

### Molecular docking of ZIKV NS5

Molecular docking was performed using the Molecular Operating Environment (MOE, 2010.10, Chemical Computing Group Inc., Montreal, QC, Canada) software with default parameters. The protein structure of the ZIKV MR766 strain NS5 (PDB: 5TFR) [39] has been used, and the docking site has been defined by the MOE Site Finder functionality. The results of docking were generated with the method of Triangle Matcher (Placement) and ASE (Rescoring). The optimal geometric conformation of the best result was selected from the Ligand Interactions feature.

### Expression and purification of ZIKV NS5, RdRp, and DENV NS5 protein

The cDNA fragment of ZIKV (ZG-01 strain) NS5 (amino acids 1 to 903), RdRp (amino acids 272 to 903), or DENV2 NS5 (amino acids 1 to 900) containing an N-terminal His tag sequence was cloned into a pET-30a-mutant vector. For protein expression, the plasmids were transformed into Rosetta™ competent cells (TIANGEN BIOTECH Co., Ltd., Beijing, China), respectively. Protein expression was induced with IPTG, and cells were harvested for the purification with a Ni–NTA affinity column (His-trap HP, GE Healthcare, China) according to the manufacturer’s suggested protocol. Mass spectrometry was performed by Shanghai Applied Protein Technology Co. Ltd. (Shanghai, China). The concentration of a targeted protein was measured by SDS-PAGE using BSA (Sigma, St. Louis, MO) as a standard.

### BIAcore analysis

Surface plasmon resonance (SPR) experiments were performed in a BIAcore T100 device (BIAcore Inc., Uppsala, Sweden) using CM5 sensor chips (General Electric Company, GE) according to the protocol provided by the manufacturer. Briefly, recombinant ZIKV NS5 protein, RdRp protein, or DENV2 NS5 protein was immobilized on a CM5 chip, respectively. Different concentrations of fidaxomicin or ribavirin were injected at a flow rate of 30 μL/min for 3 min. Subsequently, data were collected for a 3-min association followed by a 20-min dissociation. The chip was regenerated by injecting 10 μL of 15 mM NaOH for 20 s. All procedures were run in 1% DMSO PBS-P20 (GE) as a running buffer. The binding kinetics was analyzed with the software BIAevaluation version 3.1 using a 1:1 Langmuir binding model. The *K*_d_ was calculated as previously described [43].

### Polymerase activity assay

ZIKV NS5 polymerase activity was determined using a primer extension reaction with a fluorescently labeled RNA primer/RNA template complex (P/T) as described by Lu et al. [46]. Briefly, fluorescently labeled oligonucleotide primer (Cy5-RNA) and unlabeled RNA template were mixed and then slowly cooled down for annealing. A typical primer extension reaction was performed by ZIKV NS5 polymerase at various concentrations, followed by incubation for 2 h at 37 °C. To determine the inhibitory activity of fidaxomicin, the polymerase reactions were initiated by addition of fidaxomicin at gradient concentrations ranging between 0 and 200 μM. The products were detected by denaturing polyacrylamide gel electrophoresis (Urea PAGE), and the results were scanned using an Odyssey infrared imaging system (LI-COR Biosciences, Lincoln, NE). The images were analyzed, and proper RNA bands were quantified using Image J software version 1.52a (NIH, Bethesda, MD, USA).

### Luciferase reporter assays

293T cells were cultured in 24-well plates (~ 1 × 10^5^ cells per well) and co-transfected with pISRE-Luc (100 ng) and the plasmid coding for RIG-I (100 ng) together with the ZIKV NS5 plasmid at indicated concentrations. Control pcDNA3.1 vector was added to ensure that the same total amount of DNA was used for each transfection. To normalize for transfection efficiency, 5 ng of the constitutive Renilla luciferase reporter pRL-TK (Promega, San Luis Obispo, CA, USA) was added to each transfection. Six hours after transfection, cells were treated by fidaxomicin at indicated concentration for 30 h, followed by analysis of cell lysates for luciferase activity using the Dual-Luciferase Reporter Assay System Kit (Promega) according to the manufacturer’s protocol.

### In vitro antiviral assay

The anti-ZIKV activity of fidaxomicin and ribavirin was evaluated in vitro by plaque assay. Various cell lines, including SNB19 (6 × 10^4^/well, 10% FBS DMEM), A172 (7 × 10^4^/well, 10% FBS DMEM), Vero (6 × 10^4^/well 10% FBS DMEM), Huh7 (7 × 10^4^/well, 10% FBS DMEM), HUVECs (7 × 10^4^/well, 15% FBS M199 medium supplemented with 2 mM l-glutamine, 0.02 mg/mL endothelial cell growth supplement, and 0.05 mg/mL heparin), and A549 (6 × 10^4^/well, 5% FBS DMEM), were plated in 12-well plates and allowed to attach overnight, followed by infection with ZIKV (ZG-01 strain or MR766 strain, respectively, at indicated MOI) in the presence of different concentrations of fidaxomicin, ribavirin, or vehicle control for 1 h, followed by two washes with 1 × PBS and incubated with fresh medium (without virus) consisting of corresponding concentrations of the compounds for further 48 h. The cell supernatants were collected to quantify ZIKV infection by plaque assay as previously described [47] or by RT-qPCR. The 50% effective concentration (EC_50_) and 90% effective concentration (EC_90_) were estimated by plotting values from triplicate independent experiments using GraphPad (non-linear fit, log inhibitor vs normalized response). The anti-DENV activity of fidaxomicin was evaluated in vitro by RT-qPCR.

### Cell viability assay

Cell viability was detected by the 3-(4,5-dimethyl-2-thiazolyl)-2,5-diphenyl-2H-tetrazolium bromide (MTT) assay according to a previously described method [48]. Briefly, cells were seeded in 96-well plates at a density of 1 × 10^4^ cells per well and cultured at 37 °C for 24 h, followed by exposure to the serially diluted fidaxomicin or ribavirin separately for 48 h. Cell survival rate was determined by MTT reduction assay according to the manufacturer’s protocol (Invitrogen). The 50% cytotoxic concentration (CC_50_) was defined as the compound concentration required to inhibit the cell growth by 50% compared with the control assay. The assay was performed in triplicates in three independent experiments.

### RNA extraction, reverse transcription, and real-time PCR

RNA from cell supernatants or from mouse tissues (brain and testis) was prepared with RaPure Viral RNA/DNA Kit (Magen, Guangzhou, China) according to the manufacturer’s instructions. The first-strand cDNA was synthesized using random hexamer primers with the Revert Aid Reverse Transcription Kit (Thermo Fisher Scientific, Rockford, IL, USA), and real-time PCR was carried out by using LightCycler® 480 Probes Master (2× reaction mix buffer including DNA polymerase) (no. 04887301001, Roche, Basel, Switzerland). Virus concentration was determined by interpolation onto an internal standard curve made up of a 7-point dilution series of in vitro transcribed RNA and tissue weight to quantify ZIKV RNA as copies/gram tissue. Primer sets used for real-time PCR are shown in Additional file 1: Table S1. Reverse transcription and real-time PCR were performed for at least three times.

### Western blotting analysis

Tissue specimens obtained from mice or cells were lysed with RIPA lysis buffer (Millipore, Bedford, MA) containing a cocktail of protease and phosphatase inhibitors (Sigma-Aldrich, St. Louis, MO, USA). Protein concentrations were measured with a bicinchoninic acid (BCA) protein assay (Thermo Fisher Scientific, Rockford, IL, USA). Protein samples were separated by sodium dodecyl sulfate-polyacrylamide gel electrophoresis (SDS-PAGE) and transferred onto a polyvinylidene difluoride (PVDF) membrane. Non-specific antibody binding sites were blocked with 5% non-fat milk in Tris-buffered saline (TBS) (20 mM Tris-HCl [pH 7.6], 135 mM NaCl, and 0.1% Tween 20) for 1 h at room temperature and then incubated with the following primary antibodies: anti-STAT2 (1:1000, sc166201, Santa Cruz Biotechnology, Santa Cruz, CA, USA), anti-ZIKV E (1:1000, BF-1176-56, BioFront Technologies, Tallahassee, USA), anti-ZIKV NS5 (1:1000, BF-6A1-100UG, BioFront), and anti-ZIKV NS3 (1:1000, GTX133309, GeneTex, Alton Pkwy, Irvine, CA, USA). Anti-GAPDH (1:2000, ab181602, Abcam, Cambridge, MA, USA) or mouse anti-GAPDH (1:1000, ab9484, Abcam) was used as a loading control. Membranes were incubated with horseradish peroxidase-conjugated secondary antibody, and signals were detected by enhanced chemiluminescence using a commercial kit (Thermo Fisher Scientific) according to the manufacturer’s suggested protocols. The relative protein level was quantified by densitometry and normalized to GAPDH or mGAPDH level using Image J software (NIH, Bethesda, MD, USA).

### Animal experiments

For ZIKV infection and antiviral treatment studies, mice were randomly assigned to groups (*n* = 12–13/group). Male and female 5-week-old *Ifnar1*^−/−^ mice were infected subcutaneously (s.c.) with ZIKV (ZG-01 strain, 2 × 10^4^ PFU for each mouse) 1 h before administration of treatment agents. Compounds were prepared in sterile pharmaceutical dosage form of 1:10:89 (DMSO : Tween80 : 5% glucose solution) less than 4 h prior to administration in mice. Fidaxomicin was administered by tail vein injection daily at 20 or 10 mg/kg for 7 days. The identity of the treatment groups was blinded to the researcher administering treatments. Survival, disease signs, and the weight of each mouse were recorded on day 0 and then every other day from 1 to 15 days post-infection (dpi). Mice were humanely euthanized if they could no longer right themselves or were unresponsive to stimuli. Tissues, including the brains and testes, were collected from each animal. Part of each tissue was fixed in 4% paraformaldehyde or in PBS for at least 24 h prior to paraffin embedding and sectioning for use in immunohistochemistry analysis, immunofluorescence analysis, or analysis of viral RNA. Total RNA was extracted from tissues, and ZIKV RNA was quantified as described above.

### Tissue immunofluorescence and immunohistochemistry

Immunofluorescence assay was carried out on paraffin-embedded mouse brain and testis tissue slides, incubated with an anti-ZIKV E antibody (1:500, BioFront Technologies, Tallahassee, FL, USA). The secondary antibody was Rhodamine Red™-X (RRX) AffiniPure F (ab')_2_ Fragment Goat Anti-Mouse IgG (1:500, AB_2338781, Jackson ImmunoResearch Laboratories, Inc. West Grove, PA, USA), and 4-,6-diamidino-2-phenylindole (DAPI, 1:1000, Sangon Biotech, Shanghai, China) was used to stain the nuclei. The immunofluorescent images were taken with an inverted microscope (Carl Zeiss, Oberkochen, Germany). For histologic staining, the fixed tissues were dehydrated, embedded in paraffin, sectioned, rehydrated, and stained with hematoxylin and eosin (H/E). The H/E-stained sections were evaluated for viral-induced neuropathology. Unblinded histological examination was performed by a board-certified veterinary pathologist. Each slide was auto scanned by Axio Scan Z1 (Carl Zeiss, Oberkochen, Germany).

### Statistical analysis

Statistical analyses were performed on triplicate experiments using a two-tailed Student’s *t* test. The results are expressed as the mean ± standard deviation (SD).

## Results

### Fidaxomicin is predicted to bind ZIKV RdRp protein through structure-based virtual screening

**Fig. 1:**
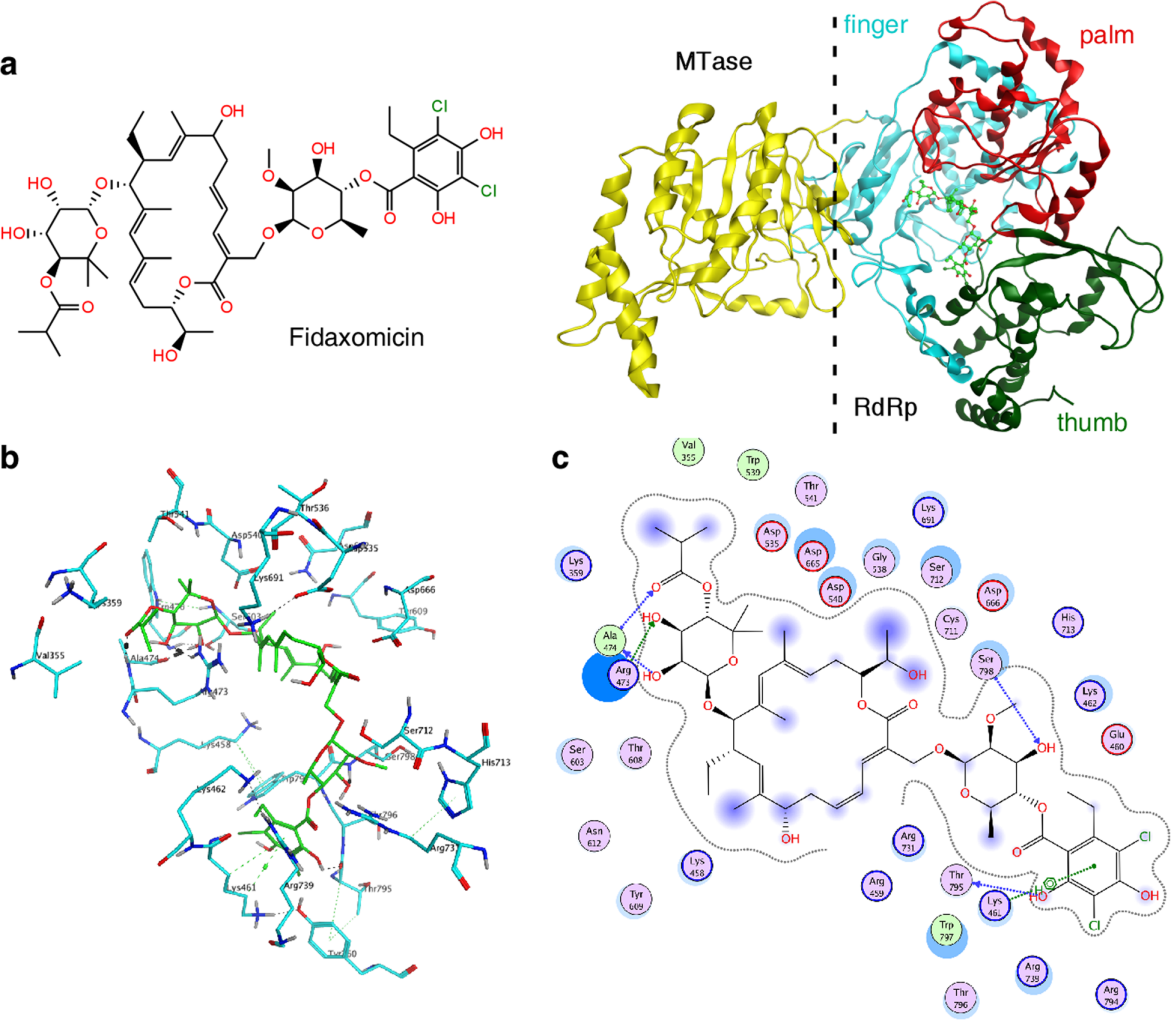
Crystal structure and binding of fidaxomicin with ZIKV RdRp. **a** The chemical structure of fidaxomicin (left) and the overview of ZIKV NS5 bound to fidaxomicin (right), displayed as ribbons with the MTase domain in yellow, palm subdomain in red, finger subdomain in cyan, thumb subdomain in olive, and fidaxomicin shown as green sticks. **b** Residues lining the pocket for fidaxomicin (green stick) are shown as cyan sticks. Individual residues are labeled according to their numbering in the ZIKV NS5 (PDB: 5TFR), H-bond interactions are indicated with black dashed lines, and H-pi-bond interactions are shown in green dashed lines. **c** Two-dimensional ligand-interaction maps of co-crystals of ZIKV NS5 domains bound with fidaxomicin were generated using Molecular Operating Environment. Polar residues are colored light purple, charged residues have an additional blue ring, and lipophilic residues are green. The degree of solvent exposure is shown by the blue halos. H-bond interactions to the amino acid side chain or main chain are shown as dashed green or blue arrows, respectively, pointing towards the H-bond acceptor. Water-mediated contacts are shown as gold dashed lines

In order to obtain anti-ZIKV candidate compounds targeting RdRp, we applied a computer docking method to simulate RdRp-compound binding and screened the compounds collection consisting of 1789 FDA-approved drugs. In brief, the crystal structure of ZIKV NS5 protein (PDB: 5tfr) (Fig. 1a) was downloaded from the Protein Data Bank website as a receptor, and the active structure of the RdRp domain was identified by the Site Finder of Molecular Operating Environment (MOE) as the docking site. Small binding molecules of ZIKV RdRp were screened in the aforementioned compounds collection by employing the ASE Rescoring function and the Triangle Matcher placement method. In the structure-based virtual screening, fidaxomicin received the highest score and was predicted to bind ZIKV RdRp domain with 5 H-bonds and 1 H-pi-bond (Fig. 1b, c). Of note, residues putatively essential for fidaxomicin binding (795T and 798S) in the ZIKV NS5 protein are located in the priming loop (residues 787–809), an important stacking platform for the initiation of NTP entry into de novo RNA synthesis [37]. Taken together, the above results of our docking screening suggest that fidaxomicin might represent a novel inhibitor of ZIKV RdRp and ZIKV replication.

### Identification of fidaxomicin as an inhibitor of ZIKV RdRp in vitro

**Fig. 2:**
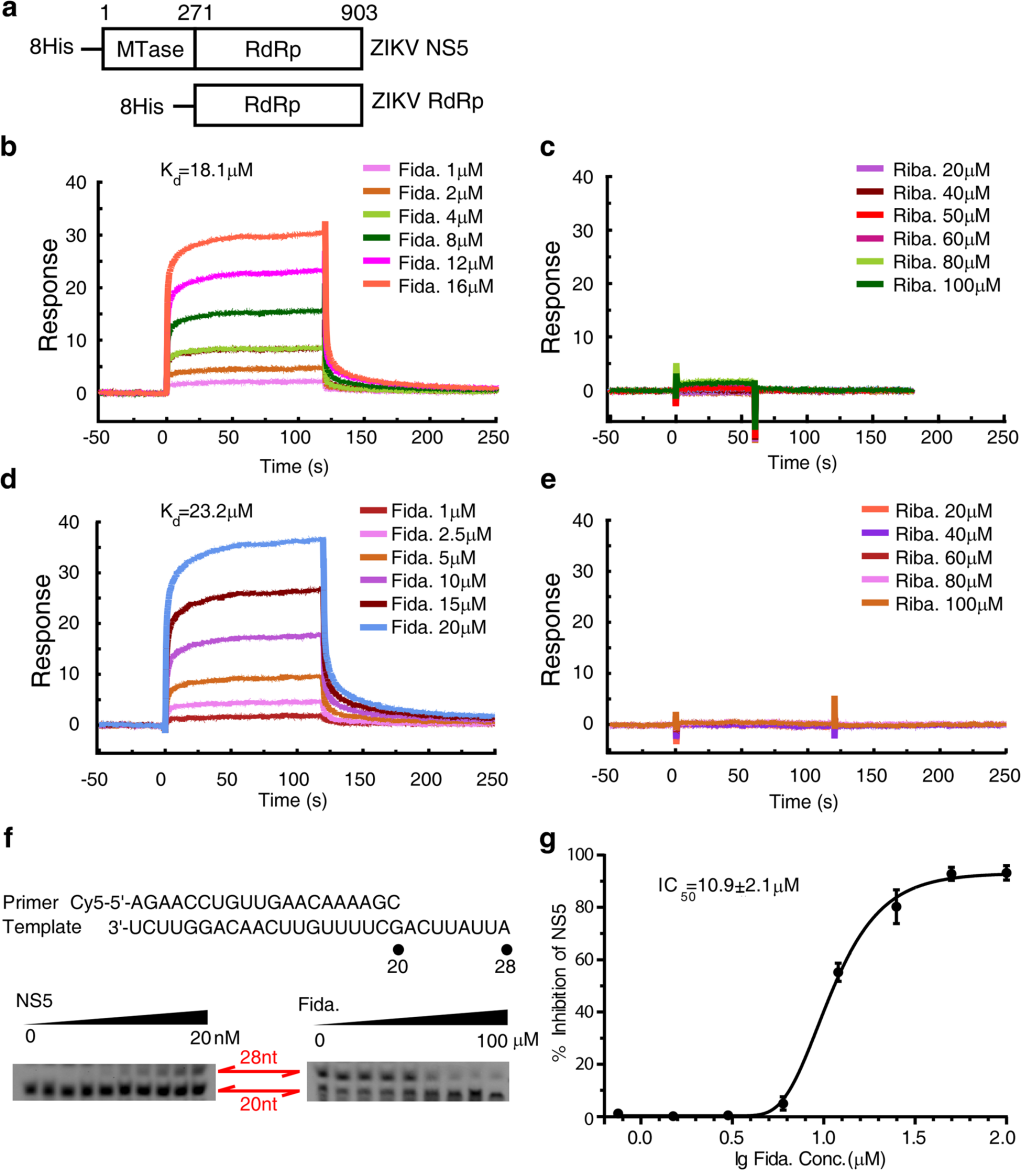
Fidaxomicin is an inhibitor of ZIKV RdRp. **a** Schematic diagram of the NS5 and RdRp expression vectors. **b–e** SPR assay to examine and characterize the binding of fidaxomicin or ribavirin to either ZIKV NS5 or RdRp, using a BIAcore T100 system. ZIKV NS5 or RdRp protein was immobilized on a CM5 chip, respectively. The analytes were consisting of serial dilutions of fidaxomicin or ribavirin, as indicated. The *K*_d_ values presented in **b** and **d** were calculated via the BIAcore T100 analysis software (BIAevaluation Version 3.1). **f** The polymerase activity of NS5 at gradient concentrations ranging between 0 and 20 nM (left) and inhibition of the polymerase activity of NS5 (20 nM) gradient concentrations of fidaxomicin ranging between 0 and 200 μM (right). Constant concentrations of Primer/Template (10 nM) were used in both assays. The red word “20 nt” indicates location of the unextended primer, and “28 nt” indicates location of the full-length product. **g** Quantification of the NS5 activities tested in **f** (right panel at bottom) using gray scale scanning by Image J. Data are representative of three independent experiments

Next, we performed experiments to investigate the biological activity of fidaxomicin for inhibition of ZIKV replication. Firstly, we prepared both full-length NS5 and a truncated NS5 containing only the RdRp domain without the MTase domain (Fig. 2a). These two forms of ZIKV RNA polymerase were expressed in and purified from *Escherichia coli* and were used for subsequent experiments. Our results of SPR assay using the BIAcore analysis showed that fidaxomicin directly bound ZIKV NS5 full-length protein (*K*_d_ value = 19 μM) (Fig. 2b), in contrast to the very weak interaction shown between ribavirin and the NS5 protein (Fig. 2c). Moreover, a strong, clear binding was also observed between fidaxomicin and ZIKV RdRp protein (Fig. 2d), further indicating that the binding was indeed between fidaxomicin and the RdRp enzyme domain ZIKV NS5. Notably, the binding between ribavirin and the RdRp protein was weak (Fig. 2e), further suggesting a specific interaction between fidaxomicin and RdRp. To investigate whether binding of fidaxomicin to the RdRp alters the biochemical function of NS5 RdRp, we assessed the polymerase activity of NS5 with or without treatment with fidaxomicin using an in vitro non-radioactive primer extension assay [46]. As shown in Fig. 2f, g, fidaxomicin was able to inhibit the RNA polymerase activity of NS5, whereas in the absence of fidaxomicin, the NS5 protein was capable of synthesizing the 28-nt viral RNA products in a dose-dependent manner. Consistently, the inhibitory effect of fidaxomicin on ZIKV RdRp-catalyzed RNA synthesis was also dose-dependent (Fig. 2f), and the IC_50_ value was estimated to be 10.9 μM as quantified by the amount of the RNA products (Fig. 2g).

Moreover, as it was reported that ZIKV NS5 targets IFN-regulated transcriptional activator STAT2 and mediates STAT2 degradation [30], we subsequently examined whether fidaxomicin could interrupt STAT2 degradation induced by NS5 and further suppress the antagonism of ZIKV on IFN signaling. We co-transfected 293T cells with the expression vector containing ZIKV NS5 and RIG-I(N), which is well known to activate the signaling pathway for IFN1, together with pISRE-Luc and increasing concentration of fidaxomicin. Our results demonstrated that overexpression of ZIKV NS5 inhibited the reporter luciferase activity driven by RIG-I(N), but fidaxomicin did not liberate the suppression of IFN signaling induced by ZIKV NS5 (Additional file 1: Fig. S1a). Furthermore, the results of western blotting of the expression of STAT2 showed that fidaxomicin did not prevent the degradation of STAT2 in the presence of NS5 either (Additional file 1: Fig. S1b). Taken together, these data suggest that the inhibition of NS5 by fidaxomicin is mainly achieved through direct binding to the RdRp active site, which is independent of the antagonistic effect of NS5 on IFN signaling.

### Fidaxomicin suppresses ZIKV infection in vitro

**Fig. 3:**
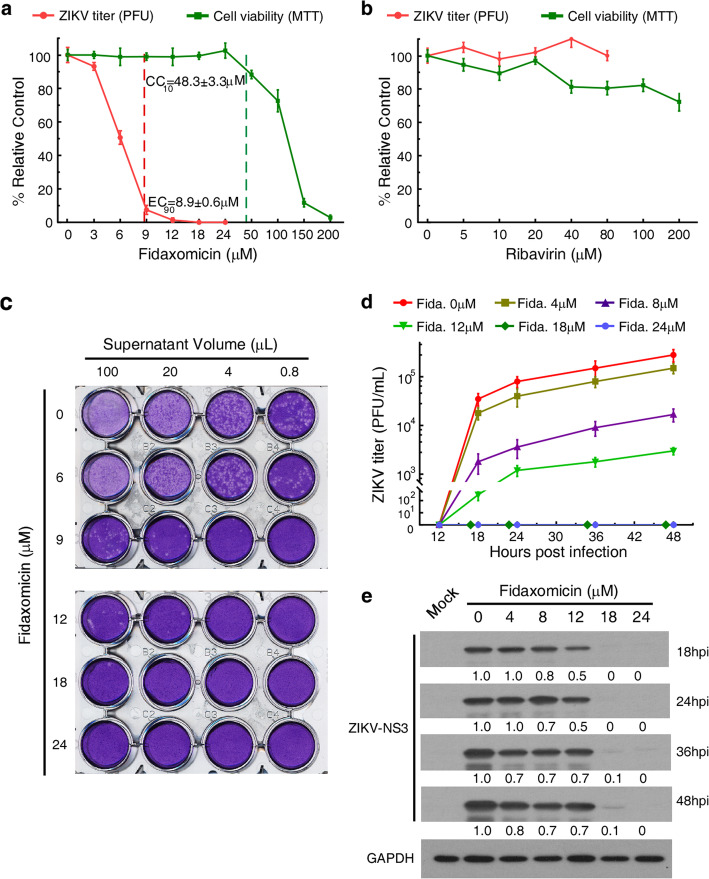
Fidaxomicin blocks ZIKV infection in vitro. **a** Antiviral and cytotoxicity spectrum of fidaxomicin in SNB19 cells. ZIKV (ZG-01) titer (the red line) was quantified by plaque assay, and cell viability (the green line) was detected by 3-(4,5-dimethylthiazol-2-yl)-2,5-diphenyltetrazolium bromide (MTT) assay. The values of EC_90_ and the CC_10_ are marked respectively. The data are the mean values ± standard deviation from triplicate experiments. **b** Antiviral and cytotoxicity spectrum of ribavirin in SNB19 cell line. ZIKV (ZG-01) titer (the red line) was quantified by plaque assay, and cell viability (the green line) was detected by MTT assay. The data are the mean values ± standard deviation from three independent experiments performed in triplicate. **c** Representative images of plaque assay. The ZIKV (ZG-01) titer in the supernatant was detected by plaque assay on new monolayers of Vero 76 cells. The supernatant was obtained from SNB19 cells culture treated with fidaxomicin at indicated concentrations at 48 h post-infection (hpi). **d** The anti-ZIKV activity of fidaxomicin in SNB19 cells. Vero cells were infected with the supernatant obtained from SNB19 cells culture treated by fidaxomicin at indicated concentrations, and the ZIKV (ZG-01) titers were detected by plaque assay at indicated hpi. Data are representative of three independent experiments performed. **e** Western blotting analysis of protein expression of ZIKV NS3 in the cell lysates of SNB19 for the anti-ZIKV activity of fidaxomicin at indicated hpi

Next, we employed plaque reduction assay and RT-qPCR to evaluate the antiviral effects of fidaxomicin on ZIKV (ZG-01 and MR766 strains) infection in a variety of host cell types, including SNB19, A172, A549, Huh7, Vero, and HUVECs. The results showed that fidaxomicin was effective against multiple strains of ZIKV, including Asian and African strains in various types of host cells (Table 1 and Additional file 1: Table S2). In the light that neurodegenaration could be involved in ZIKV pathogenesis in the CNS, we tested the antiviral effects of fidaxomicin and ribavirin in CNS cells. Notably, compared with that of ribavirin, the EC_50_ value of fidaxomicin was lower by 10-fold in SNB19 cells (Table 1). In addition, fidaxomicin also showed low EC_90_ value (approximately 9 μM, shown in Table 1), relative to the CC_10_ value (about 50 μM) in SNB19 cells (Fig. 3a), indicating that fidaxomicin possessed strong anti-ZIKV activity without significant cell cytotoxicity against the host cells. By contrast, ribavirin was far less effective against ZIKV infection in SNB19 cells under the non-cytotoxic concentration (Fig. 3b). Interestingly, as recent studies reported that ribavirin can inhibit ZIKV infection in cell culture [49, 50], our results showed that compared to ribavirin, fidaxomicin possessed higher anti-ZIKV activity and lower cellular toxicity. Furthermore, as shown in Fig. 3c, d, the inhibitory effect of fidaxomicin on ZIKV production was dose-dependent, and ZIKV infection was completely undetectable when the concentration of fidaxomicin exceeded 18 μM, strongly suggesting a dose-dependent suppressive effect of fidaxomicin treatment on ZIKV multiplication. To confirm the antiviral activity at higher MOI of virus, we performed the antiviral assays with MOI 1.0, 2.0, and 4.0 of ZIKV and quantified the viral loads cultured in SNB19 cells by PFU assay and RT-qPCR. Our results showed that the obtained EC_50_s (shown in Additional file 1: Fig. S2 a and b) were similar to those shown in Table 1. Moreover, we chose ZIKV NS3, NS5, and E protein to quantify the viral loads in response to fidaxomicin treatment. As shown in Fig. 3e, the amount of ZIKV NS3 was decreased by fidaxomicin treatment in a time-dependent manner. Similar observation that ZIKV NS5 and E proteins were also decreased by fidaxomicin treatment was made via western blotting analysis (Additional file 1: Fig. S2 c and d).

**Table 1:** In vitro efficacy of fidaxomicin and ribavirin against 2 different ZIKV strains in 4 different cell lines

ZIKV strain	Cell line	Fidaxomicin			Ribavirin		
		EC_50_ (μM)^a^	EC_90_ (μM)^b^	CC_50_ (μM)^c^	EC_50_ (μM)^a^	EC_90_ (μM)^b^	CC_50_ (μM)^c^
ZG-01	Vero	11.7 ± 2.1	48.2 ± 5.3	126.0 ± 10.3	> 100	> 100	485.5 ± 20.1
	SNB19	6.0 ± 1.0	8.9 ± 0.6	113.7 ± 9.9	> 100	> 100	478.2 ± 17.2
	Huh7	7.7 ± 1.7	17.8 ± 3.5	76.4 ± 5.8	12.7 ± 2.3	54.3 ± 3.5	491.8 ± 19.4
	A549	12.2 ± 3.6	39.5 ± 4.8	135.8 ± 12.1	33.2 ± 3.4	71.6 ± 6.2	170.1 ± 25.9
MR766	Vero	12.4 ± 3.0	45.2 ± 6.2	126.0 ± 10.3	> 100	> 100	485.5 ± 20.1
	SNB19	6.8 ± 1.2	9.5 ± 1.5	113.7 ± 9.9	> 100	> 100	478.2 ± 17.2
	Huh7	7.3 ± 2.5	16.2 ± 3.9	76.4 ± 5.8	15.6 ± 2.1	58.3 ± 3.2	491.8 ± 19.4
	A549	13.0 ± 4.4	42.2 ± 4.6	135.8 ± 12.1	35.2 ± 5.2	74.6 ± 8.5	170.1 ± 25.9

^a^The 50% effective concentration, or the concentration necessary to reduce viral yield by 50%, was determined using a plaque assay

^b^The 90% effective concentration, or the concentration necessary to reduce viral yield by 90%, was determined using a plaque assay

^c^Concentration required to inhibit the cell growth by 50% in the absence of virus

### Fidaxomicin inhibits ZIKV replication and prevents ZIKV-infected mice from death

**Fig. 4:**
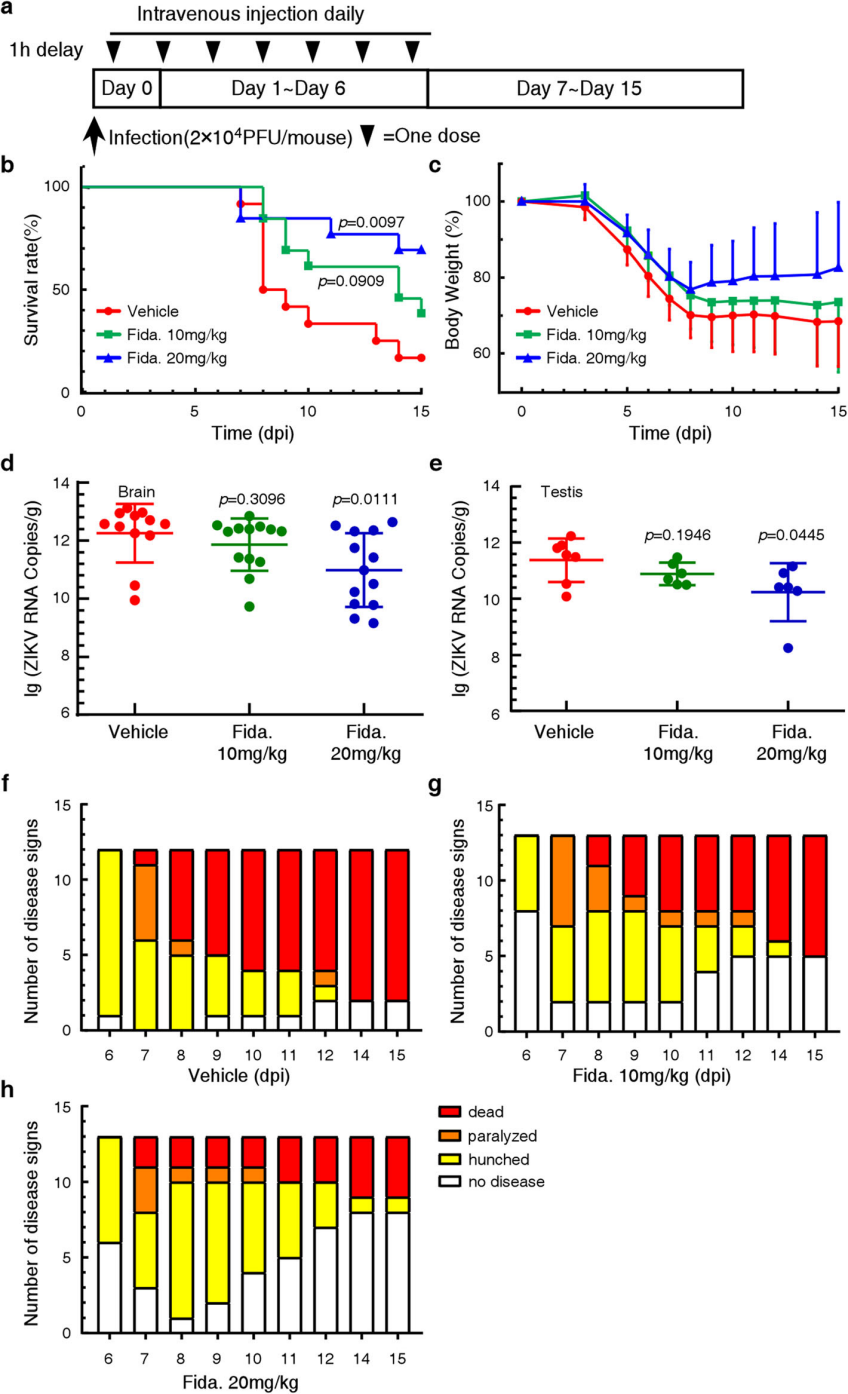
Antiviral efficacy of fidaxomicin in vivo*.***a** Schematic illustration of the animal experimental design for detecting the anti-ZIKV (ZG-01) efficacy of fidaxomicin in vivo (*n* = 12–13 animals per group). Each mouse was infected by 2 × 10^4^ PFU of ZIKV. **b** The Kaplan-Meier survival of ZIKV-infected mice treated with fidaxomicin at the indicated dosages or with control vehicle. **c** Changes in animal body weight were calculated daily for ZIKV-infected mice treated with fidaxomicin at the indicated dosages or with control vehicle. **d**, **e** Tissue viral loads in the brains (**d**) and testes (**e**) of ZIKV-infected mice were determined by RT-qPCR (*p* value, compared to the vehicle-control group). **f–h** Neurological symptoms of ZIKV-infected mice observed in the vehicle-control group (**f**) or in the fidaxomicin treatment groups (**g**, **h**) were assessed at indicated dpi. Each mouse was assigned with dead (euthanized or dead), paralyzed (dragging rear legs and/or blind), hunched (ruffled coat, hunched, and/or moving slowly), or no disease in the process of experiment. Data represent results from three independent experiments

Our in vivo experiments in mice dosed i.v. with fidaxomicin at 200 mg/kg daily for 10 days displayed no detectable adverse effect as the mice showed no significant weight loss, irreversible corneal opacities, blood abnormalities, and movement disorders (Additional file 1: Fig. S3, Table S3-S5). Given the similar EC_50_ values of fidaxomicin against Asian and African virus strains in vitro (Table 1), we selected the Asian strain (ZIKV ZG-01) to implement the subsequent animal experiments. To determine the antiviral efficacy in vivo, two doses of fidaxomicin were given i.v. daily for 7 days (Fig. 4a). Fidaxomicin improved the survival of ZIKV-infected mice in both treatment groups. Particularly, in the high-dose group, nine out of 13 mice were protected from mortality (Fig. 4b). Median survival was significantly prolonged in the fidaxomicin-treated groups, with over 15 days for the 20 mg/kg group and 14 days for the 10 mg/kg group, both of which were significantly longer than 8.5 days exhibited in the vehicle-control group. Furthermore, fidaxomicin also appeared to be able to reverse the body weight loss of ZIKV-infected mice. While all mice infected with ZIKV showed the same trend of weight loss within 7 days post-infection (dpi), between 8 and 15 dpi, an obvious protection from weight loss was observed in mice treated with 20 mg/kg/day fidaxomicin, as compared with that found in the vehicle-control group (Fig. 4c). In addition to the improvement in survival rate and weight recovery, viral RNA levels in the brain and testis on 15 dpi were significantly decreased in the two groups treated with fidaxomicin (Fig. 4d, e). A progressive decrease in the number of neurological signs, including paralysis and hunching, was observed in both treatment groups, and the degree of improvement was dose-dependent (Fig. 4f–h). Additionally, oral administration was also tested for its therapeutic efficacy, and our results showed that at the dosage as high as 225 mg/kg, orally administered fidaxomicin did not improve the outcome of ZIKV-infected mice as compared with the vehicle-control group (Additional file 1: Fig. S4).

**Fig. 5:**
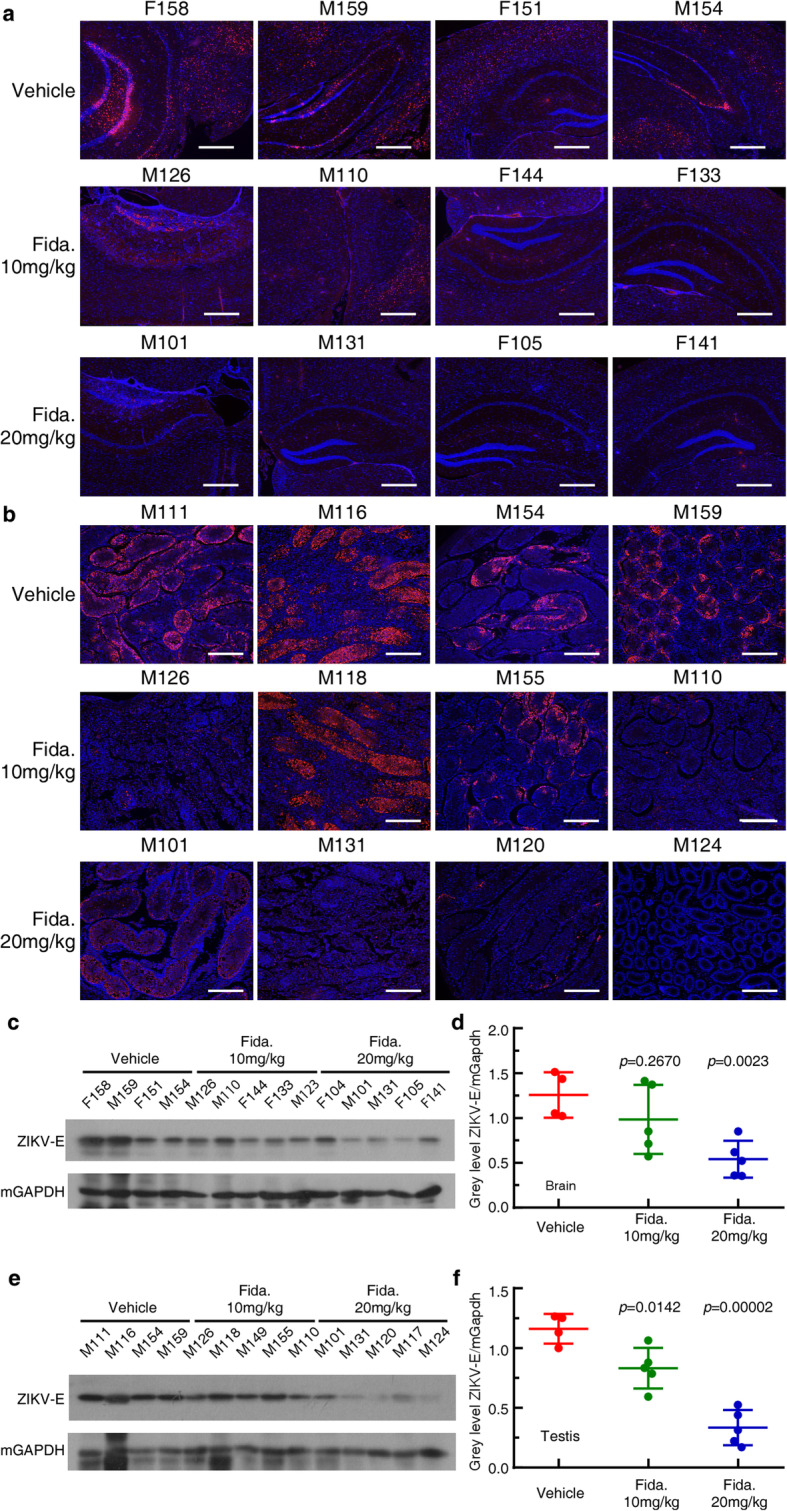
Fidaxomicin suppresses ZIKV infection in the brains and testes of ZIKV-infected mice. **a**, **b** Representative images of immunofluorescence staining for ZIKV protein (ZIKV E; red) and nuclei (with DAPI; blue) on the cerebra (**a**) and on the testes (**b**) obtained from ZIKV-infected mice treated with control vehicle or with fidaxomicin at the indicated dosages (scale bar = 200 μm). The marks, such as F158 and M159, are the unique numbers to identify each mouse during the experimental process. **c**, **e** Western blotting of the expression of ZIKV E protein in brain tissues (**c**) and in testis tissues (**e**) from ZIKV-infected mice. **d**, **f** Quantification of ZIKV E protein band intensities, normalized with mGAPDH (*p* value, compared to vehicle control)

Further, the in vivo antiviral efficacy of fidaxomicin was also detected using tissue immunofluorescence assay and western blotting analysis for the viral protein present in the brain and the testes. Immunohistochemical staining showed that as compared with the ubiquitous ZIKV-positive cells in the cerebra and testes of vehicle-control mice, fidaxomicin treatment reduced the ZIKV load in the brains and testes clearly as the results of immunostaining showed (Fig. 5a, b, Additional file 1: Fig. S5). Western blotting analysis of ZIKV E protein in the brain and testis tissues confirmed a significant suppressive effect of fidaxomicin on ZIKV in vivo (Fig. 5c–f).

**Fig. 6:**
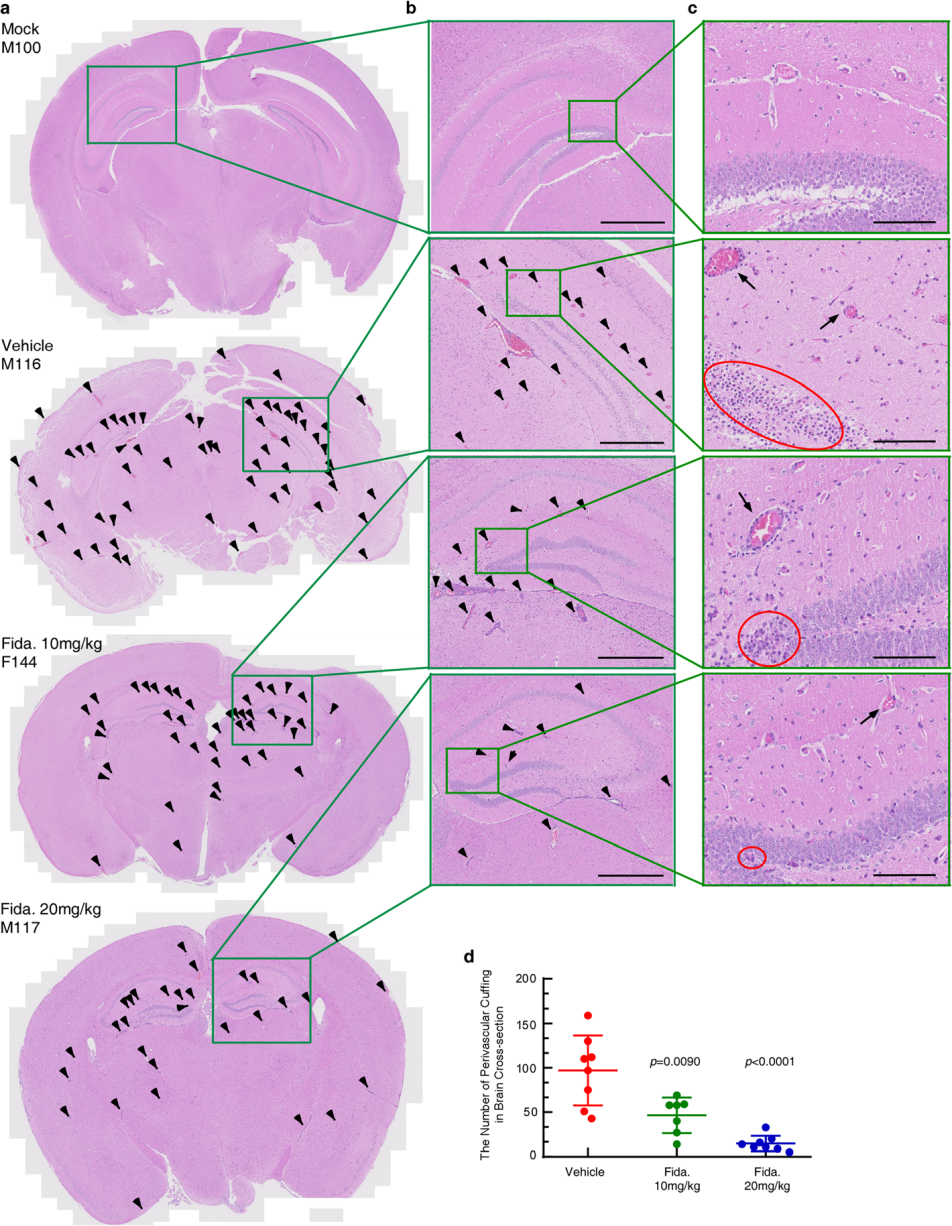
Fidaxomicin attenuates ZIKV-induced brain lesions. **a** Representative H/E staining of the cerebra specimens obtained from the mock infection group, the vehicle-control group, the 10 mg/kg fidaxomicin treatment group, and the 20 mg/kg fidaxomicin treatment group, respectively. Cerebra specimens were collected on the day of death or at the experimental endpoint. The perivascular infiltrates of mononuclear cells (“perivascular cuffing”) are indicated by black triangles. **b** Magnified images of the boxed areas in **a**, respectively. Black triangles show the perivascular cuffing; scale bar represents 500 μm. **c** Magnified images of the boxed areas in **b**, respectively. Red circles show the neuronal necrosis, and black triangles show the perivascular cuffing; scale bar represents 100 μm. **d** Quantification of the perivascular cuffing in whole cerebra tissue slices of the vehicle-control group and the fidaxomicin treatment groups (*p* value, compared to vehicle control)

To further examine whether ZIKV-induced brain lesions could be protected by fidaxomicin in vivo, histopathological and immunohistochemical analyses were performed using the cerebra specimens obtained from ZIKV-infected mice treated with two dosages of fidaxomicin. As shown in Fig. 6, multifocal histologic lesions were observed in the cerebra of vehicle-control mice, including scattered perivascular cuffing of vessels with mononuclear cells, diffused gliosis, and neuronal necrosis. In the cerebra of fidaxomicin-treated mice, the pathologic damages caused by ZIKV infection were remarkably alleviated in both treatment groups as compared with the vehicle-control group (Fig. 6a, b). Moreover, fidaxomicin therapy remarkably reduced neuronal necrosis in hippocampal pyramidal cells, as characterized by reduction of hypereosinophilic neurons and pyknosis, karyolysis, and necrotic debris (Fig. 6c). Furthermore, our data of quantitative analysis for perivascular cuffing of vessels also showed a significant decrease in both treatment groups (*p* = 0.0090 and *p* < 0.0001, respectively) (Fig. 6d), further suggesting that fidaxomicin therapy indeed protects the ZIKV-infected brains from ZIKV infection and/or ZIKV-induced pathologic changes. Collectively, our findings demonstrated a potent in vivo therapeutic efficacy of fidaxomicin against ZIKV-induced disease in virus-infected mice.

## Discussion

The key finding presented in our present report is that fidaxomicin, a currently clinically used macrocyclic antibacterial agent, is also an effective anti-ZIKV agent. Such a notion is supported by several lines of evidence provided by our study. Firstly, fidaxomicin directly binds ZIKV NS5 protein and inhibits its RdRp activity, which is essential for the replication of ZIKV genome and production of viral particles. Secondly, fidaxomicin is effective against multiple strains of ZIKV, including Asian and African strains, and potently inhibits ZIKV infection in a wide variety of cell lines of distinct tissue origins. Thirdly, fidaxomicin effectively suppresses ZIKV infection in vivo and alleviates ZIKV-associated pathological damages in infected mice. Most notably, fidaxomicin efficaciously inhibits ZIKV replication and robustly improves the survival of ZIKV-infected mice. In addition, fidaxomicin treatment of ZIKV-infected mice significantly attenuates the symptoms in the central nervous system (CNS) caused by ZIKV infection, such as paralysis and hunching. In the light that ZIKV represents a currently serious threat to global health and that fidaxomicin is already an approved clinical drug, our finding may offer a novel strategy for the control of ZIKV infection in involved individuals and the population.

It is noteworthy though that as a clinically used therapeutic agent for *C. difficile*-associated diarrhea approved by the US FDA and counterpart agencies of several other countries [51], fidaxomicin is an orally administered drug. According to pharmacokinetic studies, it is minimally absorbed through the gastrointestinal (GI) tract upon oral administration, and the resultant plasma concentration ranges within nanogram-per-milliliter scale [52]. On the other hand, as ZIKV infection is mostly a systemic disease involving multiple organs and systems [19, 53], antiviral therapy against ZIKV infection requires systematic treatment. Notably, while our animal experiments showed that orally administered fidaxomicin only weakly antagonizes ZIKV infection, presumably due to the poor GI absorption, intravenous injection of fidaxomicin exhibited potent anti-ZIKV therapeutic effect. Of particular note, the effective therapeutic doses of fidaxomicin against ZIKV (10–20 mg/kg) are much lower than the maximum non-toxic dose (> 200 mg/kg) in mice and the No Observed Adverse Effect Level (NOAEL) of the drug in rats (62.5 mg/kg) [54], providing a possibly tolerable safety profile for future application of this agent as a clinical anti-ZIKV drug. In this context, further effort in implementing pre-clinical and human testing for i.v. application of the agent is needed in future investigation. Alternatively, it is also of worth investigating whether modification of the oral dosage form of fidaxomicin could improve the intestinal absorption so that oral administration might remain effective against ZIKV infection in humans. Moreover, the design of administration employed in our current study was aimed to provide a proof-of-concept basis for future further pre-clinical as well as clinical testing. Considering the clinical characteristics of ZIKV, mostly asymptomatic infection or mild, postponing the beginning of fidaxomicin treatment to a more opportune time is certainly necessary when a more comprehensive pre-clinical trial is implemented.

The lack of drugs treating ZIKV infection, particularly those cases giving rise to newborn microencephaly, has prompted active searches for anti-ZIKV candidate compounds. Compounds identified by previous studies to possess anti-ZIKV activities include those that had been shown to be able to suppress the replication of other flaviviruses, such as sofosbuvir [55, 56], ribavirin [49], 4-HPR [57], 7-deaza-2′-*C*-methyladenosine [58], and NITD008 [59]. In addition, chemicals previously unknown to have any antiviral function were also obtained from anti-ZIKV compound searches, such as niclosamide [60] and daptomycin [23], whose mechanism and in vivo anti-ZIKV effect remain unclear. As a newly found inhibitor of RdRp, the molecular structure of fidaxomicin is remarkably distinct from those of previously identified RdRp inhibitors. Unlike traditional nucleoside or non-nucleoside RdRp inhibitors, fidaxomicin is a large macrocyclic molecule, and the residues of RdRp key to fidaxomicin binding are putatively located in a crucial functional domain of the enzyme, which might block the initiation of synthesis of ZIKV RNA. Interestingly, in the light that RdRp is fairly conserved among members of the *Flavivirus* genus, it would be worthwhile to test in future studies whether fidaxomicin also has the capacity of suppressing RdRp of other flaviviruses than ZIKV and inhibiting their replication. Indeed, our study also detected an inhibition of dengue virus by fidaxomicin (Additional file 1: Table S6) and affinity between fidaxomicin and DENV2 NS5 protein (Additional file 1: Fig. S6). Further investigation in this context is therefore needed to confirm whether the antiviral effects can be translated to other related viruses and to new flaviviruses that may emerge in the future.

## Conclusions

In summary, our findings demonstrate that a clinically in-use antibacterial drug, fidaxomicin, potently inhibits ZIKV infection both in vitro and in vivo through directly targeting an essential ZIKV non-structural protein, NS5 RdRp, robustly alleviating neurologic lesions in the CNS caused by ZIKV and preventing infected mice from morbidity. This study might provide a foundation for development of fidaxomicin as a novel anti-ZIKV drug.

## Supplementary information

**Additional file 1: Fig. S1.** Effects of fidaxomicin on ZIKV NS5-mediated interferon suppression and STAT2 degradation. **Fig. S2.** In vitro effect of fidaxomicin on ZIKV replication at various MOIs. **Fig. S3.** Changes of body weight recorded daily for mice treated i.v. with fidaxomicin at dosage of 200 mg/kg or with control vehicle. **Fig. S4.** Oral administration test for the antiviral efficacy of fidaxomicin in vivo*.***Fig. S5.** Fluorescence intensity analysis of immunohistochemical staining for ZIKV E of representative mouse tissues comparing mock and vehicle. **Fig. S6.** SPR assay to examine and characterize binding of fidaxomicin or ribavirin with DENV2 NS5. **Table S1.** Primer sequences used in this work. **Table S2.** In vitro effect of fidaxomicin on ZIKV multiplication assessed by RT-qPCR. **Table S3.** Hematological parameters of mice following a 10-day treatment with fidaxomicin at dose of 200 mg/kg. **Table S4.** Biochemical parameters of mice following a 10-day treatment with fidaxomicin at dose of 200 mg/kg. **Table S5.** Relative organ weights (g/100 g of body weight) of mice following a 10-day treatment with fidaxomicin at dose of 200 mg/kg. **Table S6.** In vitro effect of fidaxomicin on DENV2 multiplication in SNB19 and A549 cells.

## Data Availability

Data are available from the corresponding authors upon reasonable request.

## Abbreviations

ZIKV: Zika virus
RdRp: ZIKV RNA-dependent RNA polymerase
NS5: Non-structural protein 5
SPR: Surface plasmon resonance
PFU: Plaque forming units
GBS: Guillain-Barré syndrome
HCV: Hepatitis C virus
DENV: Dengue virus
JEV: Japanese encephalitis virus
MTase: Methyltransferase
CDI: *Clostridium difficile* infection
CBCAS: The Cell Bank of the Chinese Academy of Sciences
ATCC: American Type Culture Collection
FBS: Fetal bovine serum
STR: Short tandem repeat
SYSU: Sun Yat-sen University
pISRE: IFN-stimulated response element
pISRE-Luc: IFN-stimulated response element-luciferase reporter construct
MOE: The Molecular Operating Environment
dpi: Days post-infection
DAPI: 4-,6-Diamidino-2-phenylindole
H/E: Hematoxylin and eosin
SD: Standard deviation
PDB: World Protein Data Bank
CNS: The central nervous system
NOAEL: The No Observed Adverse Effect Level
STAT2: Signal transducer and activator of transcription 2
