# Supplementary material for: Antibiotic fidaxomicin is an RdRp inhibitor as a potential new therapeutic agent against Zika virus
Source: BMC Med. 2020 Jul 31;18:204. doi: 10.1186/s12916-020-01663-1 (PMC7392643; doi:10.1186/s12916-020-01663-1)
Supplement: Supplementary file 1 — Additional file 1: Fig. S1. Effects of fidaxomicin on ZIKV NS5-mediated interferon suppression and STAT2 degradation. Fig. S2. In vitro effect of fidaxomicin on ZIKV replication at various MOIs. Fig. S3. Changes of body weight recorded daily for mice treated i.v. with fidaxomicin at dosage of 200 mg/kg or with control vehicle. Fig. S4. Oral administration test for the antiviral efficacy of fidaxomicin in vivo.Fig. S5. Fluorescence intensity analysis of immunohistochemical staining for ZIKV E of representative mouse tissues comparing mock and vehicle. Fig. S6. SPR assay to examine and characterize binding of fidaxomicin or ribavirin with DENV2 NS5. Table S1. Primer sequences used in this work. Table S2. In vitro effect of fidaxomicin on ZIKV multiplication assessed by RT-qPCR. Table S3. Hematological parameters of mice following a 10-day treatment with fidaxomicin at dose of 200 mg/kg. Table S4. Biochemical parameters of mice following a 10-day treatment with fidaxomicin at dose of 200 mg/kg. Table S5. Relative organ weights (g/100 g of body weight) of mice following a 10-day treatment with fidaxomicin at dose of 200 mg/kg. Table S6. In vitro effect of fidaxomicin on DENV2 multiplication in SNB19 and A549 cells. [file 12916_2020_1663_MOESM1_ESM.docx]

**Supplementary Information for**

**Antibiotic fidaxomicin is an RdRp inhibitor as a potential new therapeutic agent against Zika virus**


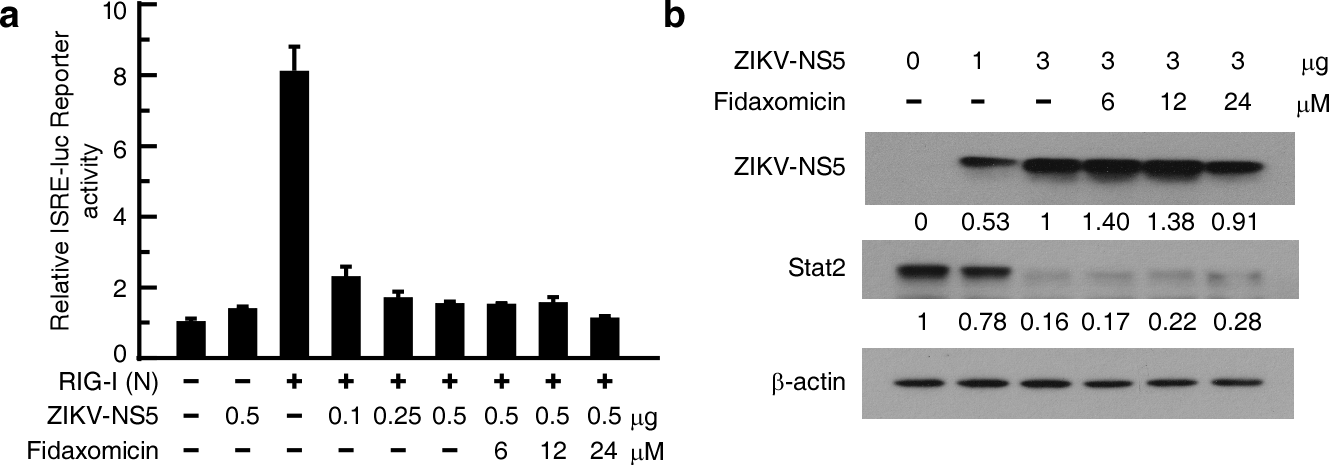


**Fig. S1.** Effects of fidaxomicin on ZIKV NS5-mediated interferon suppression and STAT2 degradation**. a** ISRE reporter assay in 293T cells transfected with increasing amounts of NS5 plasmids with or without fidaxomicin treatment. Data represent mean ± SD of three independent experiments. **b** Western blotting of the expression of STAT2 protein in 293T cells transfected with increasing amounts of ZIKV NS5 plasmids with or without fidaxomicin treatment.

**
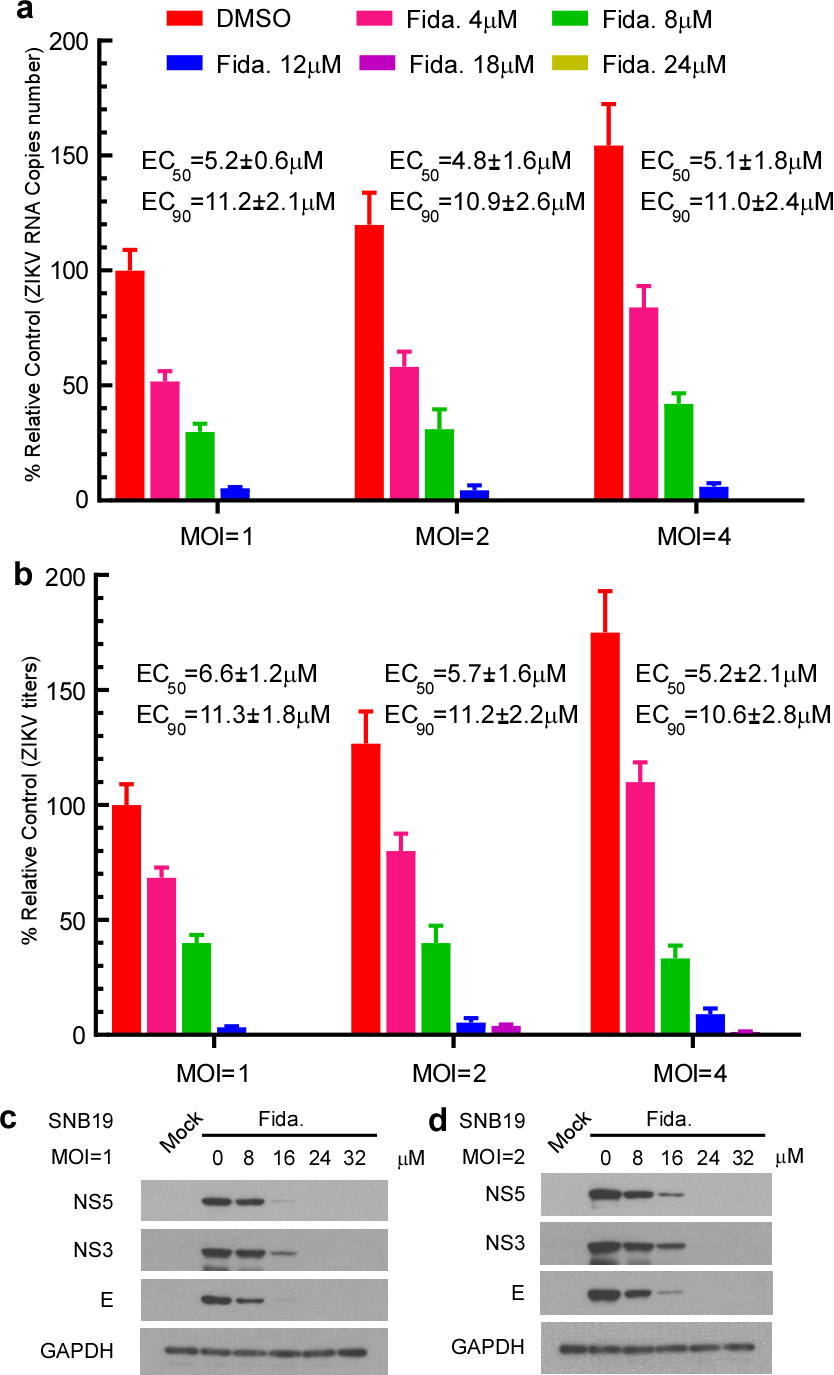
**

**Fig. S2.** *In vitro* effects of fidaxomicin on ZIKV replication at various MOIs. **a, b** *In vitro* effect of fidaxomicin on ZIKV replication with ZIKV infection at MOIs 1.0, 2.0 and 4.0 assessed by RT-qPCR (**a**) and plaque assay (**b**). **c, d** Western blotting analysis of protein expression of ZIKV NS5 and NS3in response to fidaxomicin treatment in SNB19 cells at MOI 1.0 (**c**) and MOI 2.0 (**d**).


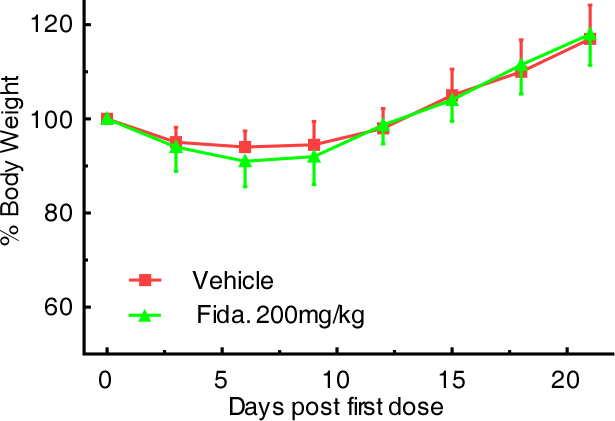


**Fig. S3.** Changes of body weight recorded daily for mice treated *i.v.* with fidaxomicin at dosage of 200mg/kg or with control vehicle.


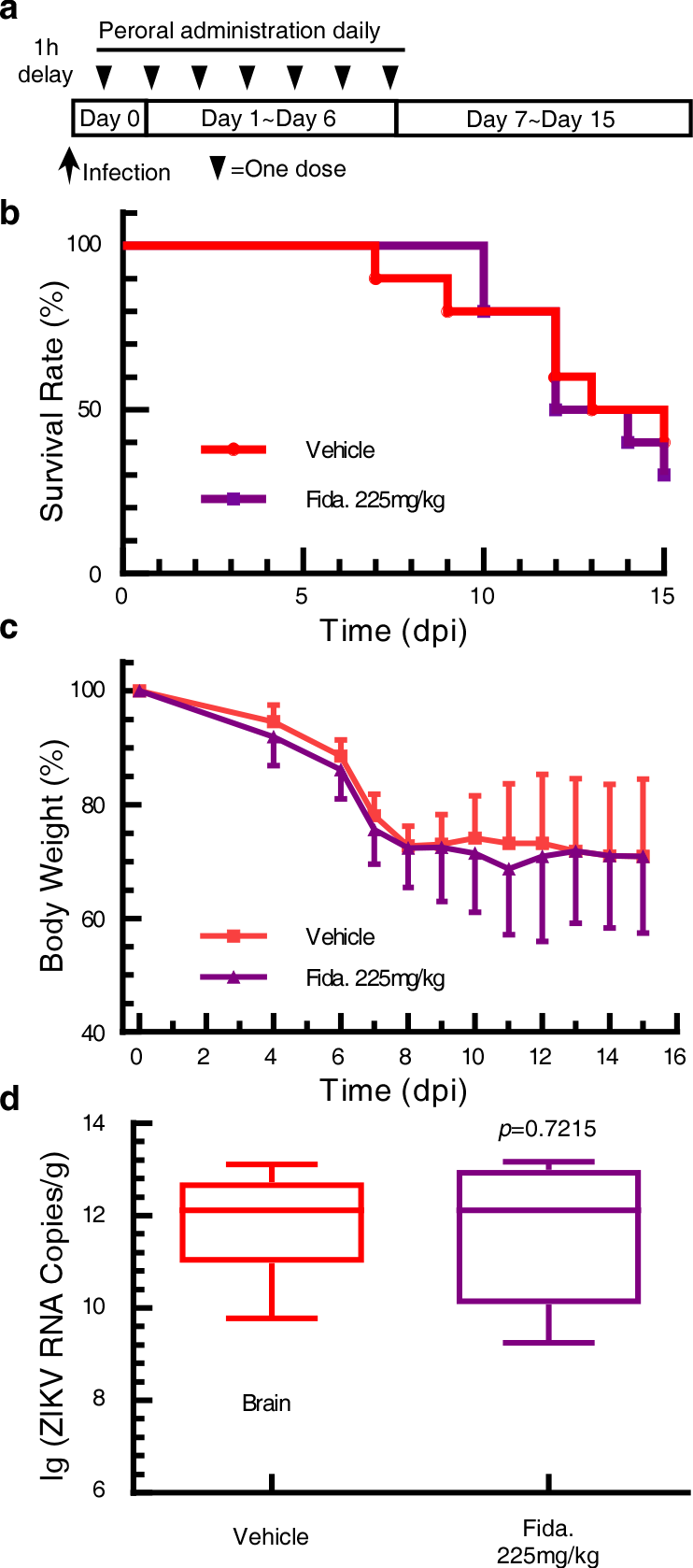


**Fig. S4.** Oral administration test for the antiviral efficacy of fidaxomicin *in vivo*. **a** Schematic illustration of the animal experimental design for the oral administration test (n=10 animals per group). **b** Kaplan–Meier survival of ZIKV-infected mice treated with fidaxomicin at the dosage of 225mg/kg. **c** Body weight changes were recorded daily for ZIKV-infected mice treated with fidaxomicin at the dosage of 225mg/kg or with control delivery vehicle. **d** Tissue viral loads in the brains of ZIKV-infected mice were determined by RT-qPCR (*P* value, compared to vehicle control).


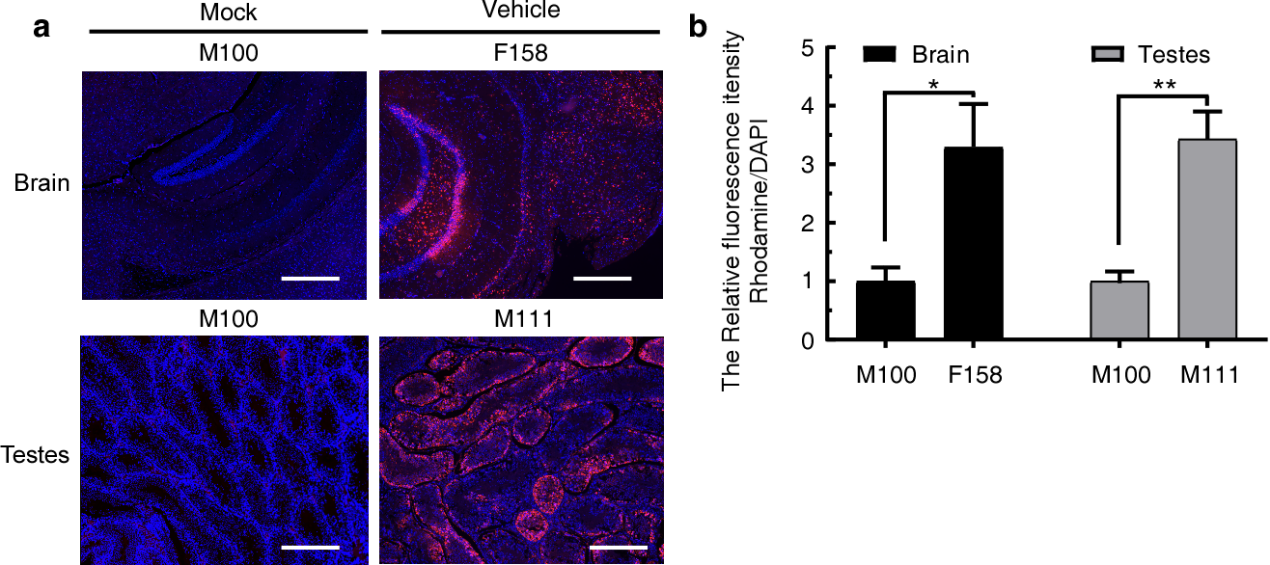


**Fig. S5.** The fluorescence intensity analysis of immunohistochemical staining for ZIKV E of representative mouse tissue between mock and vehicle.  **a** Representative image of immunofluorescence staining for ZIKV protein (ZIKV E; red) and nuclei (with DAPI; blue) on the cerebra and on the testes obtained from ZIKV-infected mice treated with Mock (no infected) or with Vehicle (infected ZIKV). (scale bar = 200 μm). The marks, such as F158, M111, are the unique numbers to identify each mouse during the experimental process. **b** Quantification of ZIKV E protein in the indicated tissue slices. A two-tailed Student’s t-test was used for statistical analysis (*, *P* < 0.05, **, *P*<0.01).


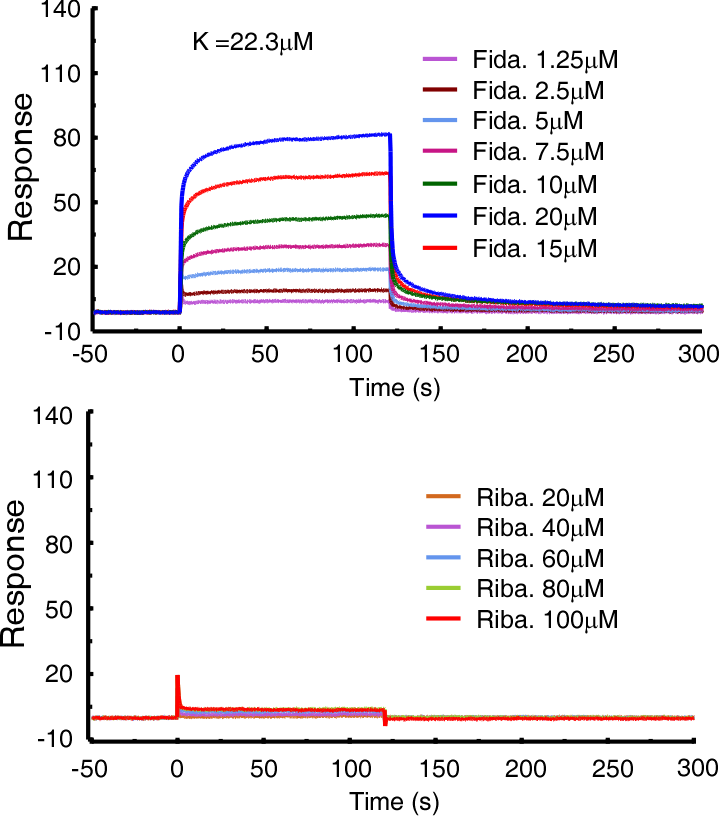


**Fig. S6.** SPR assay to examine and characterize binding of fidaxomicin or ribavirin with DENV2 NS5.

**Table S1. Primer sequences used in this work**

|  | Primers | Sequence (5’-3’) |
| --- | --- | --- |
| ZIKV-NS5 for pcDNA3.1 | Forward | CGGGGTACCGCCACCATGCACCACCACCACCACCACCACCACGGGGGTGGAACAGGAGAGAC |
|  | Reverse | CCGGAATTCTCATTCTTCACCCAAGTAGCGAACTT |
|  |  |  |
| ZIKV-RdRp for pcDNA3.1 | Forward | CGGGGTACCGCCACCATGCACCACCACCACCACCACCACCACGAAGCTCCCAACATGAAGATC |
|  | Reverse | CCGGAATTCTCATTCTTCACCCAAGTAGCGAACTT |
|  |  |  |
| ZIKV-NS5 for pET-30a-mutant | Forward | TCCCCGCGGATGCACCATCATCATCATCATCATCATGGGGGTGGAACAGGAGAGAC |
|  | Reverse | CCGCTCGAGTTATTACAGCACTCCAGGTGTAGAC |
|  |  |  |
| ZIKV-RdRp for pET-30a-mutant | Forward | TCCCCGCGGATGCACCATCATCATCATCATCATCATGAAGCTCCCAACATGAAGATC |
|  | Reverse | CCGCTCGAGTTATTACAGCACTCCAGGTGTAGAC |
|  |  |  |
| Real-time PCR primer | Forward | CCGCTGCCCAACACAAG |
|  | Reverse | CCACTAACGTTCTTTTGCAGACAT |
|  | Probe (FAM) | AGCCTACCTTGACAAGCAGTCAGACACTCAA |

**Table S2.** ***In vitro* effect of fidaxomicin on ZIKV multiplication assessed by RT-qPCR**

| **ZIKV**  **strain** | **Cell**  **line** | **Fidaxomicin** | |
| --- | --- | --- | --- |
|  |  | **EC_50_ (μM)^a^** | **EC_90_ (μM)^b^** |
| ZG-01 | Vero | 12.5±2.2 | 52.1±5.5 |
|  | SNB19 | 9.2±2.4 | 17.3±4.8 |
|  | A172 | 8.8±1.9 | 11.8±1.4 |
|  | Huh7 | 11.1±1.6 | 21.6±1.0 |
|  | A549 | 13.0±3.3 | 17.8±5.3 |
|  | HUVECs | 14.5±1.2 | 23.2±3.0 |

**a.** The 50% effective concentration, or the concentration necessary to reduce viral RNA copy number by 50%, was determined using RT-qPCR. **b.** The 90% effective concentration, or the concentration necessary to reduce viral RNA copy number by 90%, was determined using RT-qPCR.

**Table S3. Hematological parameters of mice following a 10-day treatment with fidaxomicin at dose of 200 mg/kg^a^**

| Parameters | Vehicle | Fida. 200mg/kg |
| --- | --- | --- |
| WBC (10^9^/L) | 3.89±1.12 | 4.25±1.11 |
| Lymph# (10^9^/L) | 2.76±0.7 | 3.21±1.01 |
| Mon# (10^9^/L) | 0.13±0.09 | 0.16±0.07 |
| Gran# (10^9^/L) | 0.78±0.22 | 0.92±0.23 |
| Lymph% (%) | 75.71±10.7 | 75.75±8.94 |
| Mon% (%) | 3.4±1.01 | 3.75±0.94 |
| Gran% (%) | 16.64±5.36 | 19.82±4.42 |
| RBC (10^12^/L) | 7.01±0.73 | 7.51±1.44 |
| HGB (g/l) | 127.2±16.29 | 124.14±14.36 |
| HCT (%) | 37.95±1.54 | 38.96±2.72 |
| MCV (fl) | 51.09±2.3 | 51.61±1.84 |
| MCH (pg) | 14.72±0.44 | 14.73±0.88 |
| MCHC (g/l) | 296.89±13.05 | 302±21.82 |
| RDW (%) | 17.7±0.51 | 17.56±1.04 |
| PLT (10^9^/L) | 830.83±274.96 | 891.6±337.3 |
| MPV (fl) | 5.15±0.46 | 4.95±0.59 |
| PDW | 16.9±0.43 | 16.65±0.35 |

**a.** C57BL/6J mice (*Ifnar1*^−/−^) (n = 10 per group) were administered by gavage with fidaxomicin at doses of 200 mg/kg for 1 week and observation without fidaxomicin for 2 weeks. Data are expressed as mean ± SD. WBC: white blood cells; Lymph: lymphocytes; Mon: monocytes; Gran: granulocytes; RBC: red blood cells; HGB: hemoglobin concentration; HCT: hematocrit; MCV: mean corpuscular volume; MCH: mean corpuscular hemoglobin; MCHC: mean corpuscular hemoglobin concentration; RDW: red blood cell distribution width; PLT: platelets; MPV: mean platelet volume; PDW: platelet distribution width.

**Table S4. Biochemical parameters of mice following a 10-day treatment with fidaxomicin at dose of 200 mg/kg^a^**

| Parameters | Vehicle | Fida. 200mg/kg |
| --- | --- | --- |
| ALT (U/L) | 37.4±3.83 | 39.1±4.22 |
| AST (U/L) | 135.8±12.42 | 130.74±18.3 |
| T-BIL (μmol/L) | 19.58±2.12 | 21.14±1.87 |
| ALB (g/L) | 27.65±4.31 | 29.11±5.19 |
| γ-GT (U/L) | 2.55±0.64 | 2.75±0.81 |
| ALP (U/L) | 173.6±18.5 | 185.3±19.2 |
| TBA (μmol/L) | 12.93±1.62 | 12.75±1.54 |
| BUN (mg/dl) | 29.79±3.81 | 31.6±3.54 |
| UA (μmol/L) | 176.26±25.91 | 182.61±23.8 |
| CR (μmol/L) | 16.97±2.11 | 17.62±1.84 |
| T-CHO (mmol/L) | 2.94±1.02 | 3.12±1.33 |
| TG (mmol/L) | 1.86±0.33 | 2.11±0.45 |

**a.** C57BL/6J mice (*Ifnar1*^−/−^) (n = 10 per group) were administered by gavage with fidaxomicin at doses of 200 mg/kg for 1 week and observation without fidaxomicin for 2 weeks. Data are expressed as mean ± SD. ALT: alanine aminotransferase; AST: aspartate aminotransferase; T-BIL: total bilirubin; ALB: albumin; γ-GT: γ-glutamyltransferase; ALP: alkaline phosphatase; TBA: total bile acid; BUN: blood urea nitrogen; UA: uric acid; CR: creatinine; T-CHO: total cholesterol; TG: triglyceride.

**Table S5. Relative organ weights (g/100 g of body weight) of mice following a 10-day treatment with fidaxomicin at dose of 200 mg/kg^a^**

| Parameters | Vehicle | Fida. 200mg/kg |
| --- | --- | --- |
| Heart | 0.48±0.05 | 0.47±0.06 |
| Liver | 4.82±1.15 | 4.52±1.2 |
| Kidneys | 1.46±0.23 | 1.57±0.19 |
| lung | 0.64±0.08 | 0.66±0.09 |
| spleen | 0.23±0.13 | 0.25±0.02 |
| Testes | 0.82±0.04 | 0.86±0.03 |

**a.** C57BL/6J mice (*Ifnar1*^−/−^) (n = 10 per group) were administered by gavage with fidaxomicin at doses of 200 mg/kg for 1 week and observation without fidaxomicin for 2 weeks. Data are expressed as mean ± SD.

**Table S6. *In vitro* effect of fidaxomicin on DENV2 multiplication in SNB19 and A549 cells**

| **DENV Strain** | **Cell**  **line** | **Fidaxomicin** | |
| --- | --- | --- | --- |
|  |  | **EC_50_ (μM)^a^** | **EC_90_ (μM)^b^** |
| DENV2 (NGC) | SNB19 | 6.8±1.5 | 10.6±2.1 |
|  | A549 | 9.9±2.5 | 18.0±4.2 |

**a.** The 50% effective concentration, or the concentration necessary to reduce viral yield by 50%, was determined by RT-qPCR. **b.** The 90% effective concentration, or the concentration necessary to reduce viral yield by 90%, was determined by RT-qPCR.
